# Mechanism of Ginsenosides in the Treatment of Diabetes Mellitus Based on Network Pharmacology and Molecular Docking

**DOI:** 10.3390/ijms26115300

**Published:** 2025-05-30

**Authors:** Shengnan Huang, Fangfang Li, Dedi Xue, Xinyuan Shi, Xizhu Fang, Jiawei Li, Yuan Fu, Yuqing Zhao, Dan Jin

**Affiliations:** 1 Immunology Biology Key Laboratory, Yanbian University, Yanji 133002, China; 15053791276@163.com (S.H.); ffli@ybu.edu.cn (F.L.);; 2 Department of Immunology and Pathogenic Biology, College of Medicine, Yanbian University, Yanji 133002, China; 3 Key Laboratory of Natural Medicines of the Changbai Mountain, Ministry of Education, Yanbian University, Yanji 133002, China

**Keywords:** ginsenosides, diabetes mellitus, molecular dynamics simulation, network pharmacology, ginsenoside derivative AD-1

## Abstract

Diabetes mellitus (DM) is a multifactorial metabolic disorder characterized by chronic hyperglycemia and systemic metabolic dysregulation. Although ginsenosides, the primary bioactive components of *Panax ginseng* Meyer, exhibit regulatory effects on glucose and lipid metabolism, their precise mechanisms and key targets in DM remain incompletely understood. Unlike previous studies focusing solely on crude extracts or individual ginsenosides, this study integrates network pharmacology, molecular docking, and molecular dynamics (MD) simulations to systematically elucidate the multi-target mechanisms of ginsenosides, with experimental validation using the ginsenoside derivative AD-1. Network pharmacology identified 134 potential targets, with protein–protein interaction (PPI) analysis revealing 25 core targets (such as NFKB1, HDAC1, ESR1, and EP300). Molecular docking and MD simulations showed that ginsenosides have stable binding conformations with these targets and exhibit excellent dynamic stability. Notably, in vivo experiments using AD-1 in streptozotocin-induced type 1 diabetic mice confirmed its therapeutic efficacy, significantly downregulating key diabetic markers (e.g., NFKB1 and HDAC1) in pancreatic tissues—a finding unreported in prior studies. This study not only revealed the multitarget pharmacological mechanism of ginsenosides but also highlighted the therapeutic potential of AD-1. These findings provide a foundation for further mechanistic studies and suggest new strategies for the application of novel ginsenoside derivatives in diabetes therapy.

## 1. Introduction

Diabetes mellitus (DM) is a heterogeneous metabolic disorder characterized by chronic hyperglycemia, arising from insufficient insulin secretion and/or utilization due to multifactorial etiologies. Sustained hyperglycemia can progressively damage various body systems, such as the eyes, kidneys, heart, blood vessels, and nerves, ultimately leading to functional impairment or organ failure. In more severe cases, it may trigger acute metabolic disorders such as diabetic ketoacidosis (DKA) and hyperosmolar hyperglycemic syndrome. According to the statistics of the World Health Organization (WHO), diabetes has over 100 known complications [1]. These complications collectively contribute to the growing global burden of diabetes. In recent years, there has been a dramatic increase in the prevalence and incidence of diabetes worldwide. The International Diabetes Federation (IDF) reported that there were 537 million diabetes patients globally in 2021, with an expected rise to 578 million by 2030 and 700 million by 2045 [2]. DM now ranks just behind cardiovascular disease and cancer as a major global public health concern. The classification of diabetes is based on the understanding of its pathophysiology, etiology, and clinical manifestations. With ongoing research, these classifications are expected to evolve further. According to the WHO diabetes expert committee, DM is classified into four major types: type 1 diabetes mellitus (T1DM), type 2 diabetes mellitus (T2DM), other specific types of DM, and gestational diabetes mellitus (GDM) [3]. Among them, T2DM accounts for approximately 90–95% of all diabetes diagnoses [4].

The islet β cells synthesize and secrete insulin, which enters the bloodstream and regulates intracellular metabolic processes throughout the body. When the function of islet β cells is damaged and the insulin secretion becomes absolutely or relatively deficient, the blood glucose levels rise, eventually resulting in diabetes. In addition, studies have shown that genetic and environmental factors are also involved in the onset of diabetes and identified some risk factors closely related to the development of diabetes, such as infection [5], obesity [6], age [7], and gestational diabetes history [8]. Due to incomplete understanding of diabetes pathogenesis and the lack of fully effective treatments, there is a growing interest in exploring new therapeutic strategies for diabetes. With advancements in research and technology, it has been discovered that traditional Chinese medicine (TCM) exhibits characteristics such as multi-target actions, low toxicity, and minimal side effects. Furthermore, studies have demonstrated that ginsenosides [9], Scutellaria baicalensis [10], and other TCM herbs can effectively regulate blood glucose levels and may serve as adjunctive therapies in the clinical management of diabetes.

**Figure 1:**
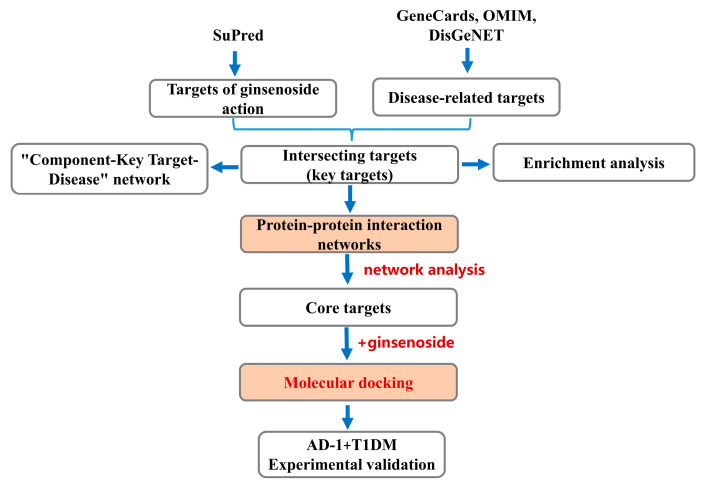
Schematic representation of the study design and analysis workflow. Potential targets of ginsenosides were predicted using SuPred, while diabetes-related targets were collected from GeneCards, OMIM, and DisGeNET databases. Intersecting targets were identified and subjected to enrichment analysis. Protein–protein interaction networks were constructed using the STRING database and visualized with Cytoscape (versions 3.7 and 3.10), with core targets selected based on topological parameters (degree, betweenness, closeness). Molecular docking was performed between ginsenosides and the top five core targets, and findings were further validated in an in vivo AD-1 intervention experiment using STZ-induced T1DM mice.

Ginseng, a perennial herb of the Araliaceae family, possesses a complex chemical composition and exhibits numerous biological activities, as well as unique pharmacological effects. Modern pharmacological studies have revealed that ginseng exhibits multiple pharmacological properties, including immunomodulatory, anti-inflammatory, anti-tumor, antioxidant, and anti-apoptotic effects [11]. The effective components in ginseng include ginsenosides (the main active component), polysaccharides, polypeptides, flavonoids, volatile oils, and amino acids [12]. Based on their molecular structures, ginsenosides can be categorized into three main types: protopanaxadiol (PPD), protopanaxatriol (PPT), and oleanolic acid type (OA) [13]. Previous studies have shown that ginsenosides, in addition to their anti-inflammatory and neuroprotective effects, inhibit tumor cell growth and modulate the tumor microenvironment [14,15]. Recent evidence suggests that ginsenosides play a variety of roles in the treatment of diabetes and its complications, such as protecting islet β cells, promoting glucose absorption in peripheral tissues, and improving insulin resistance through antioxidant, anti-apoptotic, and anti-inflammatory mechanisms. For example, ginsenoside Rb1 can maintain the metabolic balance of diabetic KKAy mice by reversing the disturbance of intestinal flora [16], and alleviate the cardiac dysfunction caused by hyperglycemia, hyperlipidemia, and abnormal levels of adipocytokines [17]. Ginsenoside Rg1 can effectively control hyperglycemia induced by T2DM through antioxidant and anti-inflammatory mechanisms [18] and prevent cognitive dysfunction in T2DM mice by inhibiting the PLC-CN-NFAT1-signaling pathway [19]. To systematically investigate the therapeutic targets and mechanisms of ginsenosides in diabetes treatment, we applied a network pharmacology-based data-mining approach. Moreover, we verified the therapeutic effect of the ginsenoside derivative AD-1 in STZ-induced T1DM mice and observed downregulation of key target proteins (e.g., NFKB1 and HDAC1) in the mouse pancreas, thus providing a basis for innovative applications of ginsenosides in the treatment of diabetes (Figure 1).

## 2. Results

### 2.1. Screening of Potential Targets of Ginsenosides Against Diabetes

**Figure 2:**
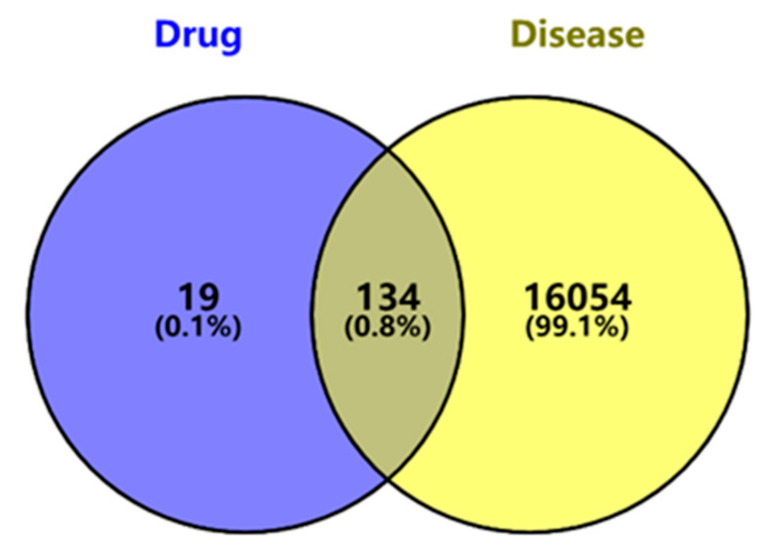
Venn diagram showing the overlap between predicted targets of ginsenosides and diabetes-related genes. A total of 134 overlapping target proteins were identified by intersecting the predicted targets of ginsenosides (blue, derived from SuPred) and diabetes-related genes (yellow, derived from GeneCards, OMIM, and DisGeNET databases). The intersection was visualized using VENNY2.1, and these 134 common targets were used for subsequent network pharmacology and enrichment analyses.

The PubChem database was utilized to retrieve the SMILES notation of ginsenosides (CC(=CCC(C)([C@H]1CC[C@@]2(C1CC[C@H]3[C@]2(CC[C@@H]4[C@@]3(CC[C@@H] (C4(C)C)O)C)C)C)C)O)C). Subsequently, the SuPred database was employed to predict the target genes of ginsenosides, resulting in a total of 153 target genes after refinement. Disease-related targets associated with “Type 1 diabetes mellitus” and “Type 2 diabetes mellitus” were collected from GeneCards, OMIM, and DisGeNET databases, and they were subsequently merged. Target names were standardized using the UniProt database, converting UniProt IDs to corresponding gene symbols. The targets retrieved from the three databases were combined and de-duplicated, resulting in 16,188 targets related to the two types of diabetes. The VENNY2.1 tool was then used to generate a Venn diagram based on the obtained ginsenosides targets and diabetes-related genes (Figure 2). A total of 134 intersections were identified as potential candidate targets of ginsenosides for diabetes treatment.

### 2.2. Protein–Protein Interaction (PPI) Network Analysis and Screening of Key Targets

**Figure 3:**
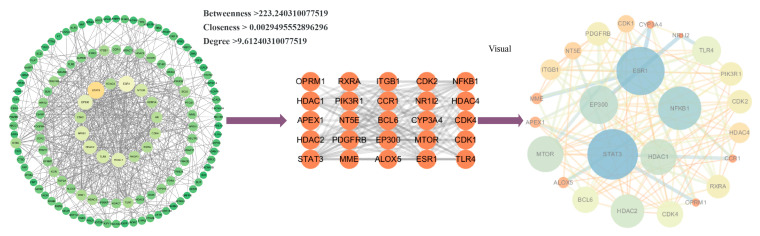
Construction and analysis of the PPI network for ginsenoside-associated diabetes targets. Nodes represent proteins, and edges represent interactions (the highest confidence score was set to 0.9, constructed using the STRING database and visualized with CytoScape 3.10). Core targets were selected based on three network topological parameters: betweenness > 223.240310077519, closeness > 0.0029495552896296, degree > 9.61240310077519. The top five targets (by degree centrality) are labeled: STAT3 (degree = 49), ESR1 (degree = 45), EP300 (degree = 39), NFKB1 (degree = 36), and HDAC1 (degree = 36).

**Figure 4:**
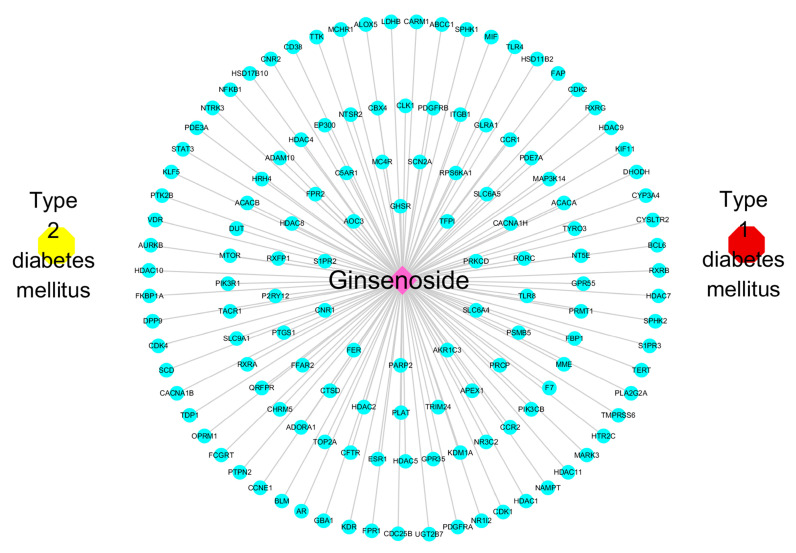
Drug-key target-disease network. Network visualization of the relationships among ginsenoside, key target genes, and diabetes subtypes (T1DM and T2DM). The network was constructed and visualized using Cytoscape. The central pink node represents ginsenoside, surrounding blue nodes represent 134 intersecting key targets, and yellow and red symbols represent T2DM and T1DM, respectively. Edges indicate predicted associations between ginsenoside and its target genes, as well as between target genes and diabetes subtypes.

A total of 134 key targets were input into the STRING database to construct the PPI network for Homo sapiens, with a threshold set to “highest confidence score was set to 0.9”. The network was visualized in Cytoscape (versions 3.7 and 3.10; Figure 3), resulting in 129 nodes and 620 edges. Nodes represent protein products, and edges indicate PPI. Twenty-five key targets were selected based on the following criteria: betweenness > 223.24, closeness > 0.00295, and degree > 9.61. Among these, the top five targets were STAT3 (degree: 49), ESR1 (degree: 45), EP300 (degree: 39), NFKB1 (degree: 36), and HDAC1 (degree: 36). Finally, CytoScape 3.10 software was used to construct the “component-target-disease” regulatory network consisting of 137 nodes and 134 edges (Figure 4). In summary, 134 key targets associated with ginsenosides and diabetes were identified, with 25 core targets serving as central nodes in the network.

### 2.3. Gene Ontology (GO) and Kyoto Encyclopedia of Genes and Genomes (KEGG) Pathway-Enrichment Analysis

**Figure 5:**
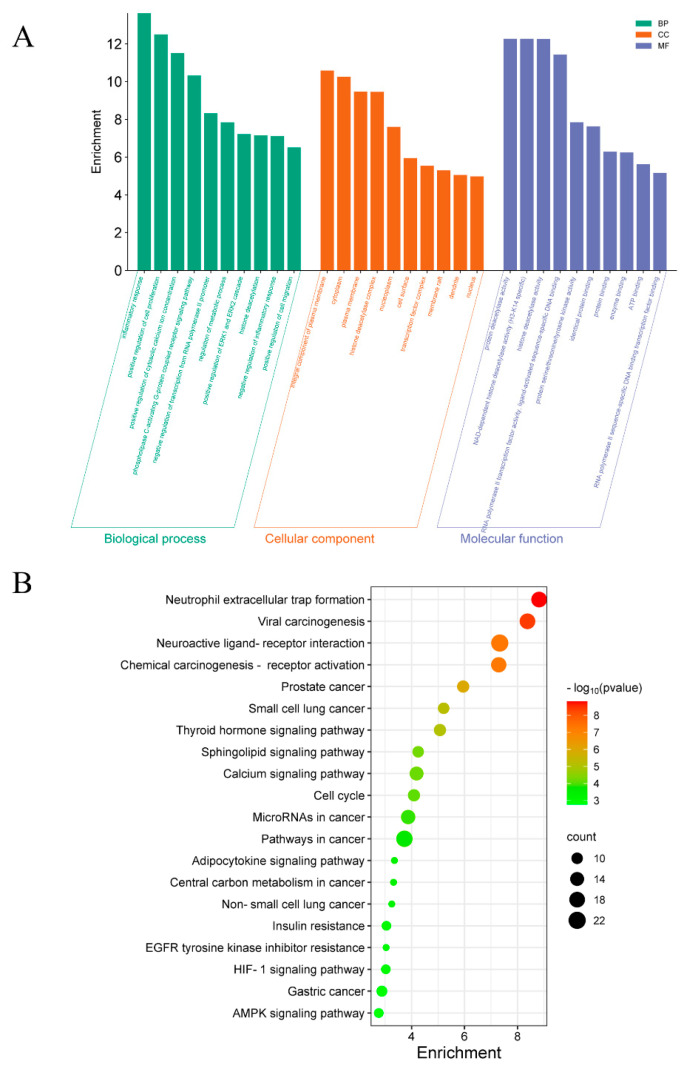
GO and KEGG pathway analysis of intersecting targets between ginsenosides and diabetes. (**A**) GO-enrichment analysis of biological processes (BP), cellular components (CC), and molecular functions (MF). The top 10 enriched terms in each category are shown. (**B**) KEGG pathway-enrichment bubble diagram. The top 20 significantly enriched pathways. Bubble size represents the number of intersecting targets in each pathway, and color gradient reflects the adjusted *p*-value.

The GO-enrichment analysis of 134 key targets was conducted using the DAVID database. Initially, gene information for “Homo sapiens” was selected, and entries related to biological process (BP), cellular component (CC), and molecular function (MF) were chosen (*p* < 0.05). As depicted in Figure 5A, the BP-enrichment analysis indicated that associated targets are involved in inflammatory response, positive regulation of cell proliferation, positive regulation of cytoplasmic calcium concentration, regulation of metabolic processes, negative regulation of the inflammatory response, and positive regulation of cell migration. The CC-enrichment analysis revealed that the target genes of ginsenoside are mainly localized to the cytoplasm, plasma membrane, nucleoplasm, cell surface, and transcription factor complexes. Furthermore, MF-enrichment analysis suggested that the target genes of ginsenoside are mainly associated with protein deacetylase activity, protein binding, enzyme binding, and ATP binding.

In order to further investigate the mechanism of action of ginsenosides in regulating signaling pathways, KEGG pathway analysis was performed in the DAVID database. The results revealed that for the treatment of diabetes mellitus, the major pathways of ginseno-sides included neuroactive ligand–receptor interactions, thyroid hormone-signaling pathway, calcium-signaling pathway, cell cycle, cancer pathway, adipocytokine-signaling pathway, insulin resistance, HIF-1-signaling pathway, and AMPK-signaling pathway, among others. The neuroactive ligand–receptor interaction is the most targeted pathway, with 22 key targets. Other pathways, such as thyroid hormone signaling, insulin resistance, AMPK signaling, and adipocytokine signaling, are also closely related to blood glucose regulation. Neuroactive ligand–receptor interactions regulate neurotransmitter systems (e.g., dopamine and serotonin) and their receptors, thereby modulating appetite and energy metabolism, which in turn affects blood glucose levels [20]. The thyroid hormone-signaling pathway regulates the basal metabolic rate, affects the synthesis and decomposition of carbohydrates, and thereby indirectly influences blood glucose levels [21]. The adipocytokine-signaling pathway regulates insulin sensitivity and energy metabolism through the secretion of cytokines, such as adiponectin and leptin by adipocytes, and is closely related to blood glucose regulation [22]. Furthermore, the pathways related to cancer and HIF-1 signaling, although having less direct connection with diabetes, may exert anti-inflammatory or antioxidant effects through common targets such as NFKB1 [23].

### 2.4. Molecular Docking of Key Target Proteins

**Figure 6:**
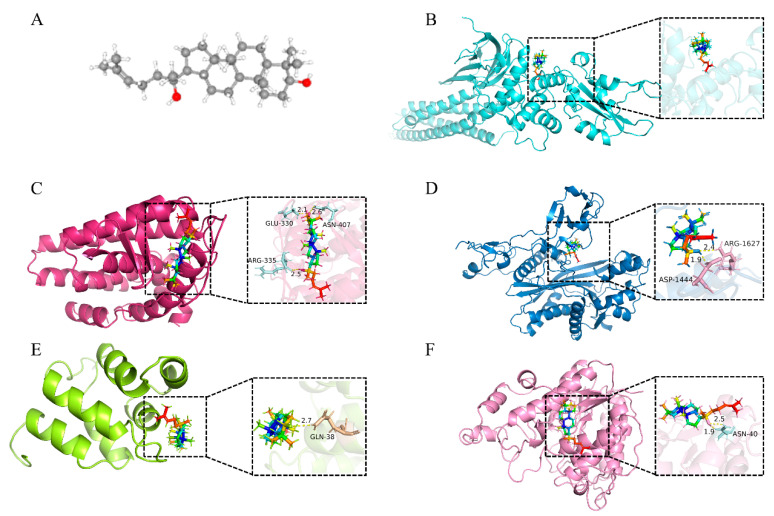
Molecular docking results. (**A**) The three-dimensional chemical structure of ginsenosides used for research and molecular docking (CAS: 74749-74-9; molecular formula: C_30_H_52_O_2_; molecular weight: 444.73 g/mol). (**B**–**F**) Predicted binding models of ginsenoside docked with STAT3 (**B**), ESR1 (**C**), EP300 (**D**), NFKB1 (**E**), and HDAC1 (**F**). The dotted lines in the diagram represent hydrogen bonds, and the text part represents the names of residues.

Based on the PPI network analysis, molecular docking experiments were conducted to evaluate the direct binding ability of ginsenosides with the top five core target proteins (STAT3, ESR1, EP300, NFKB1, and HDAC1) identified from the PPI network analysis (Figure 6 and Table 1). These targets were selected from 25 core targets based on degree centrality (reflecting the number of interactions).

Figure 6A shows the three-dimensional chemical structure of ginsenoside (CAS: 74749-74-9, molecular formula: C_30_H_52_O_2_, and molecular weight: 444.73268 g/mol). Molecular docking with STAT3 showed a docking score of −9.00 kcal/mol, but no intermolecular bonds were formed, indicating that ginsenosides may not directly bind to STAT3 (Figure 6B). Despite the lack of direct binding, STAT3 is still retained as a core target because it plays a core role in the PPI network (degree = 49, ranking first in connectivity) and participates in the insulin resistance process through signaling pathways such as insulin resistance and HIF-1 [24]. This further explains that direct binding may not be the only mechanism of action, and ginsenosides may regulate STAT3 activity through indirect interactions with other target proteins.

The docking score of ginsenoside with the target protein ESR1 was −10.25 kcal/mol, forming three hydrogen bonds with the amino acid residues ARG 335, GLU 330, and ASN 40 (Figure 6C). The docking score with EP300 was −9.19 kcal/mol, forming two hydrogen bonds with the amino acid residues ASP 1444 and ARG 1627 (Figure 6D). The docking score with NFKB1 was −8.88 kcal/mol, forming one hydrogen bond with the amino acid residue GLN 38 (Figure 6E). The docking score with HDAC1 was −9.21 kcal/mol, forming two hydrogen bonds with the amino acid residue ASN 40 (Figure 6F). The binding ability of ginsenosides with target proteins via hydrogen bonds validates the selection of diabetes-related targets and highlights the potential of ginsenosides to exert therapeutic effects through interactions with key proteins.

### 2.5. Molecular Dynamics (MD) Analysis of Complex Stability of Ginsenosides and Predicted Core Targets

**Figure 7:**
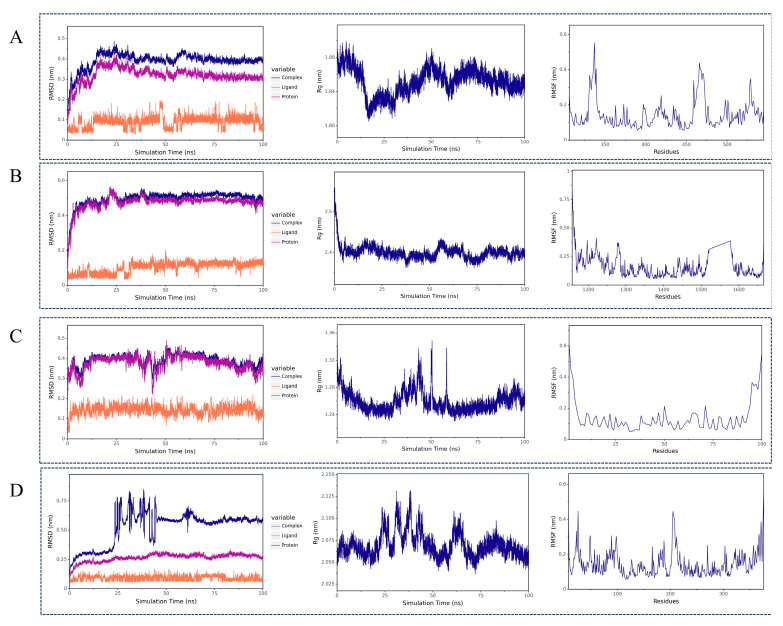
Molecular dynamics simulation of ginsenoside with four core diabetes-related target proteins. (**A**–**D**) Time evolution of RMSD, Rg, and RMSF for the complexes of ginsenoside with ESR1 (**A**), EP300 (**B**), NFKB1 (**C**), and HDAC1 (**D**) during 100 ns simulations.

Based on the molecular docking results, the complexes formed between ginsenoside and key targets (ESR1, EP300, NFKB1, and HDAC1) were selected for further molecular dynamics simulations to assess the stability of their binding conformations. Root Mean Square Deviation (RMSD) is a standard metric for quantifying structural differences between two conformations. Calculated by comparing the spatial coordinates of corresponding atoms in two molecular structures, it reflects the conformational similarity between them. In molecular dynamics simulations, RMSD tracks structural deviations relative to the initial frame, with convergence to stable values indicating system equilibration. As shown in the RMSD plots in Figure 7A–D, ginsenoside and ESR1, EP300, NFKB1, and HDAC1 complexes reached equilibrium at approximately 50 ns, 40 ns, 60 ns, and 62 ns, respectively. This showed that ginsenosides were closely bound to core targets ESR1, EP300, NFKB1, and HDAC1 during the simulation. The Radius of Gyration (Rg) curve represents the tightness of the entire protein structure. As shown in Rg in Figure 7A–D, ginsenoside and ESR1, EP300, NFKB1, and HDAC1 complexes gradually tended to be stable at 58 ns, 55 ns, 58 ns, and 70 ns Rg, respectively, indicating that the complexes maintained a compact structure during the simulations. These results further support the stable association between ginsenoside and the core targets. Root Mean Square Fluctuation (RMSF) can represent the flexibility of amino acid residues in a protein, which is the motion fluctuation of each atom or residue in a molecule during the entire molecular dynamic trajectory and is used to measure the displacement amplitude of each atom or residue relative to its average position. As shown in the RMSF plots in Figure 7A–D, multiple regions with higher flexibility were observed in the complexes, suggesting that these flexible residues may facilitate ligand binding or induce conformational changes in the core protein targets.

### 2.6. Effect of Ginsenoside Derivative AD-1 on STZ-Induced T1DM Mice and Verification of Prediction Target Proteins

**Figure 8:**
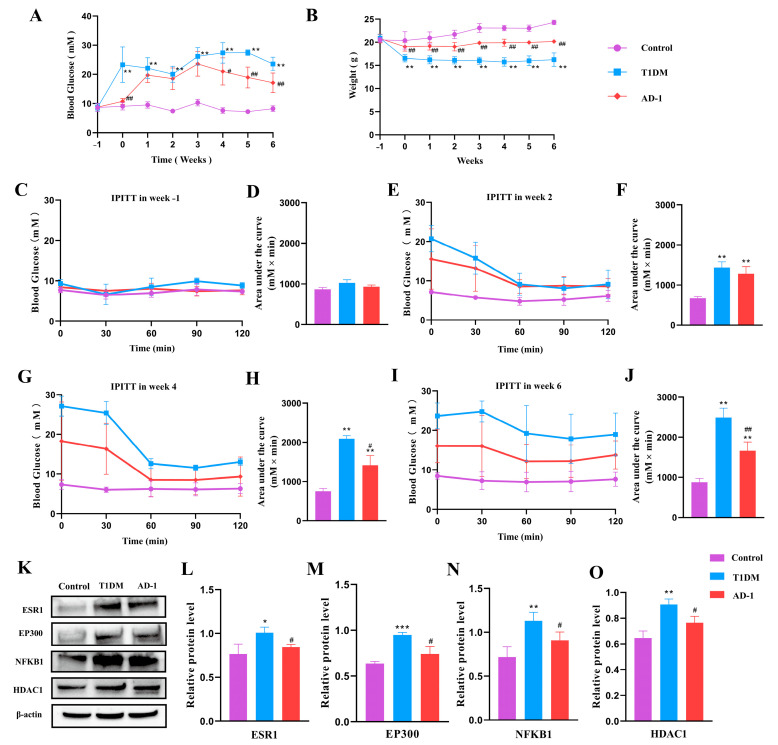
Effect of ginsenoside derivative AD-1 on STZ-induced T1DM mice and verification of the predicted target protein. (**A**) The fasting blood glucose levels of mice in each group at different time points. (**B**) The body weight of mice in each group at different time points. (**C**,**E**,**G**,**I**) IPITT at baseline (**C**), week 2 (**E**), week 4 (**G**), and week 6 (**I**). (**D**,**F**,**H**,**J**) Corresponding AUC analyses for each IPITT test. (**K**) Western blot gel images of the top four proteins for core targets. (**L**–**O**) Quantification of Western blot results for ESR1 (**L**), EP300 (**M**), NFKB1 (**N**), and HDAC1 (**O**). Data are shown as mean ± SD. Compared with control group: * *p* < 0.05, ** *p* < 0.01, *** *p*< 0.001; compared with T1DM group: # *p* < 0.05, ## *p* < 0.01 indicate significant differences.

Hyperglycemia and low body weight are key hallmarks of diabetes. To investigate the effects of AD-1 on STZ-induced T1DM, the levels of fasting blood glucose in different groups were measured weekly for 6 weeks. The results showed that the level of blood glucose in the control group was stabilized at <10 mM, and the level of blood glucose in the T1DM group was increased gradually at ≥15 mM over time and reached its highest on day 28. AD-1 administration significantly reduced blood glucose levels from day 28–42 in STZ-induced T1DM mice compared with the T1DM group (Figure 8A).

The body weight of mice in the control group increased steadily from day 0 to day 42, and AD-1 could significantly inhibit the weight loss of STZ-induced T1DM mice (Figure 8B). In the intraperitoneal insulin tolerance test (IPITT), blood glucose levels at 0 to 120 min in the T1DM group were higher than those in the control group at weeks 2, 4, and 6, and the area-under-the-curve (AUC) analysis confirmed significant glucose intolerance in the T1DM group. AD-1 treatment significantly improved glucose tolerance at weeks 2, 4, and 6, as shown by both glucose curves and AUC analysis (Figure 8C–J). In order to verify whether ESR1, EP300, NFKB1, and HDAC1 are relevant in ginsenoside treatment of diabetes, Western blot analysis was performed on pancreatic tissue from STZ-induced T1DM mice treated with AD-1 (Figure 8K–O, Supplementary Figure S1). The results showed that AD-1 treatment significantly decreased the expression of ESR1, EP300, NFKB1, and HDAC1 compared with the model group, suggesting that these proteins may be involved in the antidiabetic action of ginsenosides. These results are consistent with the network pharmacology predictions, supporting the role of these targets in ginsenoside-mediated diabetes therapy and warranting further investigation.

## 3. Discussion

At present, the management of diabetes is still focused on prevention and control. The evolution of network pharmacology has equipped researchers with a more effective and lucid methodology to elucidate the relationships between diseases and drugs. In this study, the potential targets and underlying mechanisms of ginsenosides in the treatment of diabetes were systematically analyzed by using network pharmacology approaches and various bioinformatics tools. The results revealed 134 key targets of ginsenosides in the treatment of diabetes, with 620 interrelationships among these targets. KEGG pathway-enrichment analysis indicated that these targets are mainly involved in neuroactive ligand–receptor interaction, thyroid hormone-signaling pathway, calcium-signaling pathway, cell cycle regulation, insulin resistance, HIF-1-signaling pathway, and AMPK-signaling pathway. These pathways are strongly associated with obesity, diabetes, and cardiovascular disease, suggesting that diabetes is a metabolic disorder that often coexists and interacts with other chronic diseases. In addition, in order to deeply explore the interaction mechanism between ginsenosides and core target proteins, we performed comprehensive molecular docking and molecular dynamics simulations. Docking results revealed that ginsenoside formed stable interactions with four of the top five core proteins: ESR1, EP300, NFKB1, and HDAC1. Subsequently, using MD simulations, a dynamic analysis of these protein–ligand complexes was conducted over a period of 100 nanoseconds. By monitoring the conformational changes of the complexes at the atomic level, we further verified the stability and continuity of the binding between ginsenosides and the aforementioned target proteins. This series of research results indicates that ginsenosides have the potential to regulate relevant signaling pathways and play a therapeutic role in the treatment of diabetes. However, network pharmacology analyses are inherently limited by the existing knowledge base and, thus, may overlook novel or previously uncharacterized targets and pathways.

The *ESR1* gene, located on chromosome 6q25.1, encodes estrogen receptors, which are ligand-activated transcription factors that play a key regulatory role in gene expression. *ESR1* is known to regulate cell proliferation and differentiation [25], and accumulating evidence also implicates it in the pathogenesis of diabetes [26]. A study of T2DM in Palestinian women found that PvuII and XbaI polymorphisms, which are associated with T2DM, may affect *ESR1* gene expression by altering transcription factor-binding sites within the *ESR1* gene promoter. This disruption of estrogen signaling can lead to glucose intolerance, impaired insulin sensitivity, and increased risk of T2DM [27]. Further studies have shown that both ESR1 and ESR2, members of the nuclear receptor family, can mediate the hypoglycemic effect of estrogen. Specifically, both ESR1 and ESR2 regulate blood glucose levels by altering GLUT4 content in tissues through *SLC2A4* gene-expression regulation, thereby improving diabetes conditions [28]. Taken together, the influence of the *ESR1* gene itself promotes the development of diabetes to a certain extent. However, there are relatively few studies on its mechanism of action in diabetes, which is a direction for future research.

EP300 (also known as p300) is a multifunctional protein of the histone acetyltransferase family, which interacts with other proteins based on acetylation modification activity. Alterations in EP300 expression or mutations in the *EP300* gene are closely related to the occurrence and development of diabetes. Lack of EP300 expression in islet cells can lead to a decrease in the number of pancreatic α and β cells and then cause hypoinsulinemia [29]. An epigenetic study has shown that the recruitment of EP300 is essential for effective transcription in glycotoxicity-induced transcription programs, and the knockout of Ep300 can partially prevent glycotoxicity-induced dysfunction of β cells [30]. Recent studies have proposed that Ep300 may play an important role in diabetes-related inflammation and oxidative stress through epigenetic mechanisms and transcription factor acetylation [31]. Due to the diverse roles of EP300 in diabetes pathogenesis, further studies are needed to elucidate its phenotypic and functional characteristics under pathological conditions.

Recent studies using Ingenuity Pathway Analysis (IPA) have identified the activation of multiple signaling networks involved in the progression from insulin-resistant and normal glucose tolerance individuals to T2DM patients: The NFKB1 network, as well as tumor necrosis factor, vascular endothelial growth factor, and interleukin1/1B Network [32]. Ali et al. [33] determined the expression of differential mRNAs associated with T2DM (*NFKB1*, *ZBP1*, *HSPA1B*, *TMEM173*, *DDX58,* and *CHUK*) using microarray data sets. Subsequently, the expression levels of these six mRNAs were compared in healthy people, pre-diabetic patients (pre-DM), and T2DM patients. Significantly higher expression levels of these mRNAs, including *NFKB1*, were observed in the blood of patients with prediabetes and T2DM compared to healthy controls. These findings suggest that *NFKB1* may serve as a new predictive marker and a potential therapeutic target for T2DM in patients with prediabetes. In addition, studies on epigenetic biological correlations related to inflammation, oxidative stress, and glucose metabolism disorders have found that the expression of *NFKB1* and *TIGAR* genes is upregulated in visceral fat cells of obese patients with intermittent or chronic hyperglycemia [34]. Other studies have shown that the NF-κB-signaling pathway encoded by NFKB1 plays a crucial role in the pathogenesis of T1DM. The inhibition of NFKB1 expression can suppress NF-κB activation and alleviate the associated inflammatory response [35].

*HDAC1* is a protein-coding gene that encodes proteins in the histone deacetylase /AcuC/AphA family and is a component of the histone deacetylase complex. In recent decades, the prevalence of diabetes has increased significantly. Epigenetic studies have increasingly used blood DNA methylation patterns as biomarkers for assessing diabetes risk. For example, Domingo Relloso et al. [36] identified diabetes-associated DNA methylation dysregulation in genes such as *HDAC1* and *SREBF1*, both of which are implicated in metabolic regulation and insulin sensitivity. Single-cell techniques and RNA methylation studies have revealed that crosstalk between pancreatic ductal and endocrine lineages regulates β-cell maturation and function via *METTL3*/*HDAC1*-mediated m6A RNA modification [37]. This suggests that *HDAC1* is a key marker of glucose metabolism and insulin secretion that regulates transcription during pancreatic development and endocrine differentiation through epigenetic modifications such as DNA methylation, histone modification, and non-coding RNA. In addition, transcriptome analysis of human islets and transgenic mouse islets exposed to high glucose and cytokines showed that metabolic and inflammatory stress can induce endoplasmic reticulum stress in β cells by promoting the accumulation and expression of HDAC1 on the *MCT1* promoter. This disrupts the effect of NFATc2 on beta cell function, causing β cells to dedifferentiate into an immature or dysfunctional state [38]. Inhibiting or reducing the expression of HDAC1 during metabolic or inflammatory stress can not only maintain the differentiation function of rat β cells and insulin secretion and reduce blood sugar level, but also enhance the overall antioxidant capacity of the body [39]. In recent years, HDAC inhibitors have emerged as a potential treatment for diabetes and its complications. Zhang et al. [40] used RNA sequencing to analyze the comprehensive gene-expression profile of rat islets treated with HDAC inhibitor (MS-275). This treatment upregulated genes promoting insulin secretion and reprogrammed islet gene expression, resulting in increased insulin secretion in vivo and in vitro. In addition to the use of HDAC inhibitors, direct targeting of HDAC1 may offer further potential for the treatment of diabetes and its complications. However, the therapeutic effect of ginsenosides on diabetes through HDAC1 has not been proven, and further research is needed.

The STZ-induced T1DM mouse model was used due to its rapid and well-established ability to mimic human T1DM. We assessed the expression of predicted core target proteins in pancreatic tissue following treatment with the ginsenoside derivative AD-1. The results showed that compared with the normal group, the expressions of the four core proteins ESR1, EP300, NFKB1, and HDAC1 in the pancreatic tissue of the model group were significantly increased, while the expressions of the four core proteins in the pancreatic tissue of the ginsenoside AD-1 group were markedly decreased. These results suggested that ginsenosides have potential therapeutic effects on diabetes, which provides valuable reference and research insight for future research.

Despite the promising findings presented in this study, several limitations should be acknowledged. First, the relatively small sample size in animal experiments may have limited the statistical power and generalizability of the results. Although significant differences in protein expression were observed between groups, larger-scale studies are warranted to confirm the reproducibility and robustness of these findings. Second, this research relied exclusively on a single T1DM mouse model (STZ-induced), which may not fully capture the heterogeneity and complexity of human T1DM, particularly with regard to genetic and immune-mediated subtypes. Third, while the present work identified several key target proteins (e.g., ESR1, EP300, NFKB1, and HDAC1) involved in the antidiabetic effects of AD-1, the precise molecular mechanisms underlying these regulatory interactions remain to be fully elucidated. Future studies should aim to address these gaps by incorporating multiple experimental models, expanding sample sizes, and employing advanced in vitro and in vivo approaches to dissect the mechanistic pathways in greater detail. Notwithstanding these limitations, our study provides an important foundation for further investigation into the therapeutic potential of ginsenoside derivatives in diabetes management.

## 4. Materials and Methods

### 4.1. Materials

Streptozotocin (STZ, S0130, Sigma-Aldrich, Carlsbad, CA, USA), anti-ESR1, anti-EP300, anti-NFKB1, anti-HDAC1, and anti-β-actin antibodies (Cell Signaling Technology, Boston, MA, USA), RIPA lysis buffer (R0010, Solarbio, Beijing, China), phenylmethylsulfonyl fluoride (P0100, Solarbio, Beijing, China), BCA Protein Assay Kit (P0011, Beyotime, Shanghai, China), polyvinylidene fluoride (3010040001, Roche, Shanghai, China), skim milk (P0216, Beyotime, Shanghai, China), ECL color development kit (S6009M, US EVERBRIGHT, Beijing, China), and goat anti-rabbit secondary antibody (S002, Affinity Biosciences, Melbourne, Australia).

### 4.2. Animal Experiment

Six-to-eight-week-old male C57BL/6J mice with 18–22 g were provided by Laboratory Animal Center, Yanbian University, Yanji, China. A total of 15 mice were used in the study and randomly divided into three groups: control group (*n* = 5), T1DM group (*n* = 5), and T1DM + AD-1 group (*n* = 5). The sample size (*n* = 5 per group) was determined by balancing statistical feasibility and adherence to the 3R principles (Replacement, Reduction, Refinement) for animal research. All mice were fed on a condition with 24 h light/dark cycle at 23 °C ± 2 °C and relative humidity of 45–55%. All food and water were accessed ad libitum. All animal procedures were approved by the Experimental Animal Ethical Committee of Yanbian University (approval number: YD20230065). Following a week of adaptive feeding, STZ-induced T1DM mouse model was established. Briefly, mice were injected intraperitoneally with 50 mg/kg/day STZ in 0.1 M sodium citrate buffer for 5 days. STZ solution was prepared immediately in dark place before use. After 3 days of administration, fasting blood glucose levels were measured in each group of mice. Mice with fasting blood glucose > 11.1 mM were considered diabetic and used for subsequent experiments. AD-1 (10 mg/kg) was administered by oral gavage once a day for 6 weeks to the T1DM + AD-1 group, while control and T1DM groups received an equal volume of 0.5% carboxymethylcellulose solution by oral gavage. Finally, pancreatic tissues were isolated from subsequent experiments.

### 4.3. Screening of Ginsenoside Compound Composition

The SMILES structures of ginsenosides were retrieved from the PubChem database (https://pubchem.ncbi.nlm.nih.gov/, accessed on 31 December 2023), followed by the use of the SuPred database (https://prediction.charite.de/index.php, accessed on 31 December 2023).

### 4.4. Collection and Collation of Disease Targets

Disease-related targets were collected using the keywords “Type 1 diabetes mellitus” and “Type 2 diabetes mellitus” from three major databases: GeneCards (https://www.genecards.org/, accessed on 31 December 2023) [41], OMIM (https://omim.org/, accessed on 31 December 2023) [42], and DisGeNET (https://www.disgenet.org/, accessed on 31 December 2023) [43]. The collected data were then integrated and deduplicated to obtain a comprehensive set of diabetes-associated targets.

### 4.5. Acquisition of Key Targets

The potential drug targets and diabetes-related genes were input into the VENNY2.1 website (https://bioinfogp.cnb.csic.es/tools/venny/index.html, accessed on 1 January 2024) to identify overlapping genes, which were considered as candidate targets of ginsenosides for diabetes treatment.

### 4.6. Construction of PPI Network and Screening of Core Targets

The overlapping genes identified above were imported into the STRING database (https://string-db.org/, accessed on 1 January 2024) for construction of the PPI network. Subsequently, Cytoscape software (version 3.7 and 3.10), along with Centiscape 2.2 plug-in, was utilized for visualization and development analysis of the network, nodes were sorted based on the degree algorithm, and the top five core genes were selected. The drug key target-disease network was then constructed using CytoScape 3.10 software.

### 4.7. GO and KEGG

In order to investigate the specific roles of the key target proteins of ginsenosides in the biological processes, GO-enrichment analysis was performed on the overlapping targets using the DAVID database (https://david-d.ncifcrf.gov/, accessed on 1 January 2024) [44]. Human gene information for “Homo sapiens” was selected, and entries for BP, CC, and MF were chosen. The top 10 most significantly enriched terms in each category were selected and visualized using the Bioinformatics online platform (http://www.bioinformatics.com.cn/, accessed on 6 March 2024). Additionally, KEGG pathway-enrichment analysis was performed on the overlapping targets using DAVID, and the top 20 most significantly enriched pathways were visualized using the Bioinformatics online platform.

### 4.8. Verification of Molecular Docking

The SDF files of ginsenosides were downloaded from the PubChem database and prepared by energy minimization, hydrogenation, and charge correction using Autodock Vina 1.5.7 software. The PDB format files of the core target proteins were downloaded from the PDB database (http://www.rcsb.org/pdb, accessed on 1 January 2024). Protein structures were preprocessed by removing water molecules, ligands, and crystallization ions, and by adding hydrogens and repairing missing residues using AutoDockTools. Molecular docking was then conducted using Autodock Vina software with the compound as the ligand and the protein as the receptor. Finally, PyMOL 3.0.3 software was employed to visualize the docking results.

### 4.9. MD Simulation

The MD simulations were performed using the Gromacs 2022 software package. The protein was parameterized with the AMBER14SB force field and the TIP3P water model, and small molecule ligands were parameterized using the GAFF force field. The protein–ligand complex system was constructed by merging the protein and small molecule files. All simulations were conducted under isothermal–isobaric (NPT) conditions with periodic boundary constraints. During the MD simulations, hydrogen bonds were constrained using the LINCS algorithm with an integration time step of 2 fs. Long-range electrostatic interactions were calculated using the Particle Mesh Ewald (PME) method with a cutoff distance of 1.2 nm. The non-bonded interaction cutoff was set to 10 Å, updated every 10 steps. Temperature was maintained at 298 K using the V-rescale coupling method, while pressure was controlled at one bar using the Berendsen barostat. The system underwent 100 ps of equilibration in both NVT (constant volume) and NPT (constant pressure) ensembles at 298 K, followed by a production MD run of 100 ns. Trajectory snapshots were saved every 10 ps. Finally, the simulation trajectories were analyzed using VMD and PyMOL for structural and dynamic evaluations.

### 4.10. Fasting Glucose and IPITT

After a 12 h fasting period, the blood samples were collected from the tail vein of mice in each group, and blood glucose was measured weekly with a glucose meter. In addition, each group underwent IPITT prior to model induction, as well as during the 3rd and 6th weeks post-treatment. In brief, blood glucose levels were measured at 0, 30, 60, 90, and 120 min after intraperitoneal injection of 0.5 U/kg insulin (Wanbang, Changzhou, China) in each group.

### 4.11. Western Blot Assay

Total protein was extracted from mouse pancreatic tissues and quantified using a BCA Protein Assay Kit. Equal amounts of protein were separated by SDS–PAGE, transferred onto PVDF membranes, and blocked for 1 h at room temperature. The membranes were incubated overnight at 4 °C with primary antibodies (anti-ESR1, anti-EP300, anti-NFKB1, anti-HDAC1, and anti-β-actin; CST, Danvers, MA, USA), followed by incubation with HRP-conjugated secondary antibodies for 1 h at room temperature. Protein bands were visualized using an enhanced chemiluminescence (ECL) substrate (Everbright, San Ramon, CA, USA) and detected with a chemiluminescent imaging system, as described previously [45].

### 4.12. Statistical Analysis

All experiments were performed in triplicate, and data are expressed as mean ± SD. Statistical analyses were conducted using the t-test for comparisons between two groups, or one-way ANOVA for multiple group comparisons, as appropriate. *p* < 0.05 was considered statistically significant. Analyses were performed using GraphPad Prism 10.0 (GraphPad Software, San Diego, CA, USA).

## 5. Conclusions

This research presents a comprehensive strategy for elucidating the antidiabetic mechanisms of ginsenosides by integrating network pharmacology, target prediction, PPI network analysis, and multi-level validation through molecular docking, molecular dynamics simulation, and in vivo experiments. A total of 134 potential targets and 25 core targets (such as NFKB1 and HDAC1) were identified, with metabolic pathway and target interaction analysis revealing involvement of multiple signaling networks in the antidiabetic process. In vivo studies demonstrated that AD-1 improved blood glucose levels and body weight in STZ-induced T1DM mice and downregulated key target expression in pancreatic tissues. Molecular docking and MD simulations validated stable binding of ginsenosides to core targets, confirming the accuracy of predicted interactions. In summary, our results provide a mechanistic rationale for further experimental validation and offer a strategic framework for the development of novel ginsenoside derivatives in diabetes therapy.

## Figures and Tables

**Table 1:** Docking results of ginsenosides with the top five core target proteins.

No.	Sybol	Uniprot ID	Protein Names	Degree	PDB	Docking Score (kcal/mol)
1	STAT3	P40763	Signal transducer and activator of transcription 3	49	6NJS	−9.00
2	ESR1	P03372	Estrogen receptor	45	6V8T	−10.25
3	EP300	Q09472	Histone acetyltransferase p300	39	8GZC	−9.19
4	NFKB1	P19838	Nuclear factor NF-kappa-B p105 subunit	36	2DBF	−8.88
5	HDAC1	Q13547	Histone deacetylase 1	36	6Z2J	−9.21

## Data Availability

The original contributions presented in the study are included in the article; further inquiries can be directed to the corresponding authors.

## Supplementary Materials

The following supporting information can be downloaded at: https://www.mdpi.com/article/10.3390/ijms26115300/s1.

## References

[1] Gregg E.W., Buckley J., Ali M.K., Davies J., Flood D., Mehta R., Griffiths B., Lim L.L., Manne-Goehler J., Pearson-Stuttard J. (2023). Improving health outcomes of people with diabetes: Target setting for the WHO Global Diabetes Compact. Lancet.

[2] Sun H., Saeedi P., Karuranga S., Pinkepank M., Ogurtsova K., Duncan B.B., Stein C., Basit A., Chan J.C.N., Mbanya J.C. (2022). IDF diabetes atlas: Global, regional and country-level diabetes prevalence estimates for 2021 and projections for 2045. Diabetes Res. Clin. Pract..

[3] Harreiter J., Roden M. (2023). Diabetes mellitus—Definition, Klassifikation, Diagnose, Screening und Prävention (Update 2023) [Diabetes mellitus: Definition, classification, diagnosis, screening and prevention (Update 2023)]. Wien. Klin. Wochenschr..

[4] Gora I.M., Ciechanowska A., Ladyzynski P. (2021). NLRP3 Inflammasome at the interface of inflammation, endothelial dysfunction, and type 2 diabetes. Cells.

[5] Wu R., Mumtaz M., Maxwell A.J., Isaacs S.R., Laiho J.E., Rawlinson W.D., Hyöty H., Craig M.E., Kim K.W. (2023). Respiratory infections and type 1 diabetes: Potential roles in pathogenesis. Rev. Med. Virol..

[6] Rohm T.V., Meier D.T., Olefsky J.M., Donath M.Y. (2022). Inflammation in obesity, diabetes, and related disorders. Immunity.

[7] Conrad N., Misra S., Verbakel J.Y., Verbeke G., Molenberghs G., Taylor P.N., Mason J., Sattar N., McMurray J.J.V., McInnes I.B. (2023). Incidence, prevalence, and co-occurrence of autoimmune disorders over time and by age, sex, and socioeconomic status: A population-based cohort study of 22 million individuals in the UK. Lancet.

[8] Sweeting A., Wong J., Murphy H.R., Ross G.P. (2022). A clinical update on gestational diabetes mellitus. Endocr. Rev..

[9] Liu P., Zhang Z., Cai Y., Yang Y., Yuan J., Chen Q. (2023). Inhibition of the pyroptosis-associated inflammasome pathway: The important potential mechanism of ginsenosides in ameliorating diabetes and its complications. Eur. J. Med. Chem..

[10] Wang Y., Zheng L., Liu G., Wang H. (2022). Research progress of active ingredients of scutellaria baicalensis in the treatment of type 2 diabetes and its complications. Biomed. Pharmacother..

[11] Zhou G., Wang C.Z., Mohammadi S., Sawadogo W.R., Ma Q., Yuan C.S. (2023). Pharmacological effects of ginseng: Multiple constituents and multiple actions on humans. Am. J. Chin. Med..

[12] Zhou Z., Li M., Zhang Z., Song Z., Xu J., Zhang M., Gong M. (2024). Overview of panax ginseng and its active ingredients protective mechanism on cardiovascular diseases. J. Ethnopharmacol..

[13] Piao X., Zhang H., Kang J.P., Yang D.U., Li Y., Pang S., Jin Y., Yang D.C., Wang Y. (2020). Advances in saponin diversity of panax ginseng. Molecules.

[14] You D., Hillerman S., Locke G., Chaudhry C., Stromko C., Murtaza A., Fan Y., Koenitzer J., Chen Y., Briceno S. (2021). Enhanced antitumor immunity by a novel small molecule HPK1 inhibitor. J. Immunother. Cancer.

[15] Chen Y.Y., Liu Q.P., An P., Jia M., Luan X., Tang J.Y., Zhang H. (2022). Ginsenoside Rd: A promising natural neuroprotective agent. Phytomedicine.

[16] Zhou R., He D., Zhang H., Xie J., Zhang S., Tian X., Zeng H., Qin Y., Huang L. (2023). Ginsenoside Rb1 protects against diabetes-associated metabolic disorders in Kkay mice by reshaping gut microbiota and fecal metabolic profiles. J. Ethnopharmacol..

[17] Zhang C., Han M., Zhang X., Tong H., Sun X., Sun G. (2022). Ginsenoside Rb1 protects against diabetic cardiomyopathy by regulating the adipocytokine pathway. J. Inflamm. Res..

[18] Xie Q., Zhang X., Zhou Q., Xu Y., Sun L., Wen Q., Wang W., Chen Q. (2023). Antioxidant and anti-inflammatory properties of ginsenoside Rg1 for hyperglycemia in type 2 diabetes mellitus: Systematic reviews and meta-analyses of animal studies. Front. Pharmacol..

[19] Dong X., Kong L., Huang L., Su Y., Li X., Yang L., Ji P., Li W., Li W. (2023). Ginsenoside Rg1 treatment protects against cognitive dysfunction via inhibiting PLC-CN-NFAT1 signaling in T2DM mice. J. Ginseng Res..

[20] Moon J.H., Oh C.M., Kim H. (2022). Serotonin in the regulation of systemic energy metabolism. J. Diabetes Investig..

[21] Rial-Pensado E., Canaple L., Guyot R., Clemmensen C., Wiersema J., Wu S., Richard S., Boelen A., Müller T.D., López M. (2023). Neuronal blockade of thyroid hormone signaling increases sensitivity to diet-Induced obesity in adult male mice. Endocrinology.

[22] Lustig R.H., Collier D., Kassotis C., Roepke T.A., Kim M.J., Blanc E., Barouki R., Bansal A., Cave M.C., Chatterjee S. (2022). Obesity I: Overview and molecular and biochemical mechanisms. Biochem. Pharmacol..

[23] Castillo-Rodríguez R.A., Trejo-Solís C., Cabrera-Cano A., Gómez-Manzo S., Dávila-Borja V.M. (2022). Hypoxia as a modulator of inflammation and immune response in cancer. Cancers.

[24] Kim K.E., Lee J., Shin H.J., Jeong E.A., Jang H.M., Ahn Y.J., An H.S., Lee J.Y., Shin M.C., Kim S.K. (2023). Lipocalin-2 activates hepatic stellate cells and promotes nonalcoholic steatohepatitis in high-fat diet-fed Ob/Ob mice. Hepatology.

[25] Pan P., Wen Z., Ma F., Lei Z., Pan C., Fei Q., Tian E., Wang Y., Zhu Q., Li H. (2023). Bisphenol S stimulates Leydig cell proliferation but inhibits differentiation in pubertal male rats through multiple mechanisms. Environ. Toxicol..

[26] Zhao L., Fan X., Zuo L., Guo Q., Su X., Xi G., Zhang Z., Zhang J., Zheng G. (2018). Estrogen receptor 1 gene polymorphisms are associated with metabolic syndrome in postmenopausal women in China. BMC Endocr. Disord..

[27] Ereqat S., Cauchi S., Eweidat K., Elqadi M., Nasereddin A. (2019). Estrogen receptor 1 gene polymorphisms (PvuII and XbaI) are associated with type 2 diabetes in palestinian women. PeerJ.

[28] Gregorio K.C.R., Laurindo C.P., Machado U.F. (2021). Estrogen and glycemic homeostasis: The fundamental role of nuclear estrogen receptors ESR1/ESR2 in glucose transporter GLUT4 regulation. Cells.

[29] Wild K.T., Nomakuchi T.T., Sheppard S.E., Leavens K.F., De León D.D., Zackai E.H. (2021). Hyperinsulinism in an individual with an EP300 variant of rubinstein-taybi syndrome. Am. J. Med. Genet. A.

[30] Zhang E., Mohammed Al-Amily I., Mohammed S., Luan C., Asplund O., Ahmed M., Ye Y., Ben-Hail D., Soni A., Vishnu N. (2019). Preserving insulin secretion in diabetes by inhibiting VDAC1 overexpression and surface translocation in β Cells. Cell Metab..

[31] Di Pietrantonio N., Di Tomo P., Mandatori D., Formoso G., Pandolfi A. (2023). Diabetes and its cardiovascular complications: Potential role of the acetyltransferase p300. Cells.

[32] Errafii K., Boujraf S., Chikri M. (2023). Transcriptomic analysis from normal glucose tolerance to T2D of obese individuals using bioinformatic tools. Int. J. Mol. Sci..

[33] Ali H.S., Boshra M.S., Agwa S.H.A., Hakeem M.S.A., Meteini M.S.E., Matboli M. (2022). Identification of a multi-messenger RNA signature as type 2 diabetes mellitus candidate genes involved in crosstalk between inflammation and insulin resistance. Biomolecules.

[34] Wróblewski A., Strycharz J., Oszajca K., Czarny P., Świderska E., Matyjas T., Zieleniak A., Rucińska M., Pomorski L., Drzewoski J. (2023). Dysregulation of inflammation, oxidative stress, and glucose metabolism-related genes and miRNAs in visceral adipose tissue of women with type 2 diabetes mellitus. Med. Sci. Monit..

[35] Amirshahrokhi K., Zohouri A. (2021). Carvedilol prevents pancreatic β-cell damage and the development of type 1 diabetes in mice by the inhibition of proinflammatory cytokines, NF-κB, COX-2, iNOS and oxidative stress. Cytokine.

[36] Domingo-Relloso A., Gribble M.O., Riffo-Campos A.L., Haack K., Cole S.A., Tellez-Plaza M., Umans J.G., Fretts A.M., Zhang Y., Fallin M.D. (2022). Epigenetics of type 2 diabetes and diabetes-related outcomes in the strong heart study. Clin. Epigenet..

[37] Sun J., Wang Y., Fu H., Kang F., Song J., Xu M., Ning G., Wang J., Wang W., Wang Q. (2024). Mettl3-mediated m6A methylation controls pancreatic bipotent progenitor fate and islet formation. Diabetes.

[38] Darden C.M., Vasu S., Mattke J., Liu Y., Rhodes C.J., Naziruddin B., Lawrence M.C. (2022). Calcineurin/NFATc2 and PI3K/AKT signaling maintains β-cell identity and function during metabolic and inflammatory stress. iScience.

[39] Sevastre-Berghian A.C., Ielciu I., Mitre A.O., Filip G.A., Oniga I., Vlase L., Benedec D., Gheldiu A.M., Toma V.A., Mihart B. (2020). Targeting oxidative stress reduction and inhibition of HDAC1, MECP2, and NF-kB pathways in rats with experimentally induced hyperglycemia by administration of *Thymus marshallianus* willd. Extracts. Front. Pharmacol..

[40] Zhang Y., Li M., Wang Y., Liu X., Zhou L., Zhang C., Shao L. (2020). Histone deacetylase inhibition by MS-275 potentiates glucose-stimulated insulin secretion without affecting glucose oxidation. Life Sci..

[41] Rebhan M., Chalifa-Caspi V., Prilusky J., Lancet D. (1997). GeneCards: Integrating information about genes, proteins and diseases. Trends Genet..

[42] Amberger J.S., Bocchini C.A., Schiettecatte F., Scott A.F., Hamosh A. (2015). OMIM.org: Online Mendelian Inheritance in Man (OMIM^®^), an online catalog of human genes and genetic disorders. Nucleic Acids Res..

[43] Bauer-Mehren A., Rautschka M., Sanz F., Furlong L.I. (2010). DisGeNET: A Cytoscape plugin to visualize, integrate, search and analyze gene-disease networks. Bioinformatics.

[44] Sherman B.T., Hao M., Qiu J., Jiao X., Baseler M.W., Lane H.C., Imamichi T., Chang W. (2022). DAVID: A web server for functional enrichment analysis and functional annotation of gene lists (2021 update). Nucleic Acids Res..

[45] Fang X., Lee Y.H., Jang J.H., Kim S.J., Kim S.H., Kim D.H., Na H.K., Kim K.O., Baek J.H., Surh Y.J. (2023). ARD1 stabilizes NRF2 through direct interaction and promotes colon cancer progression. Life Sci..

