# Supplementary material for: Mechanism of Ginsenosides in the Treatment of Diabetes Mellitus Based on Network Pharmacology and Molecular Docking
Source: Int J Mol Sci. 2025 May 30;26(11):5300. doi: 10.3390/ijms26115300 (PMC12155384; doi:10.3390/ijms26115300)

Supplementary Figure S1: The complete Western Blot bands of all experimental groups

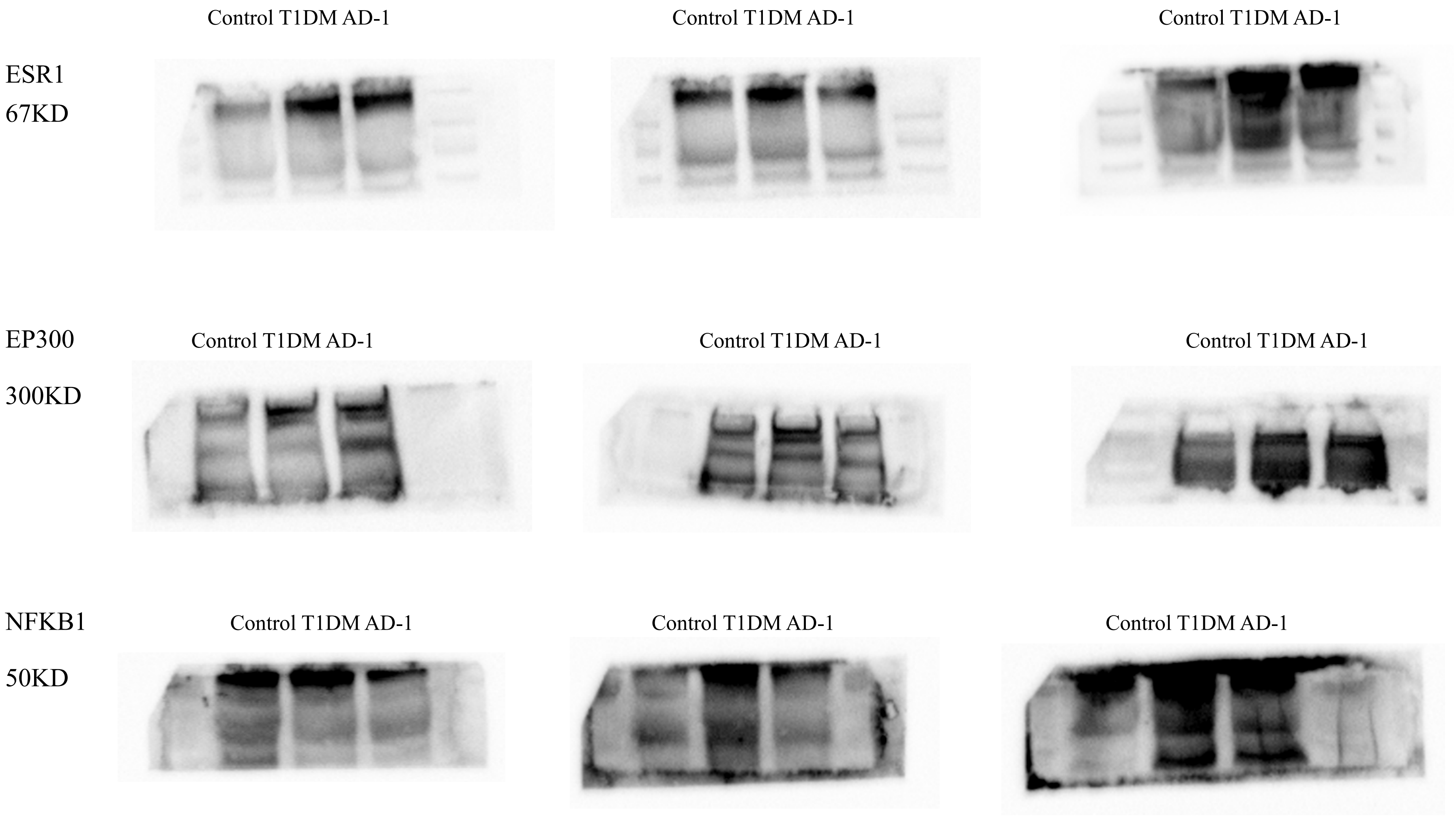

HDAC1

Control T1DM AD-1 Control T1DM AD-1 Control T1DM AD-1

55KD

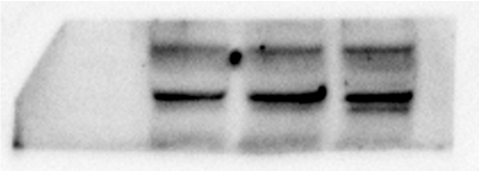

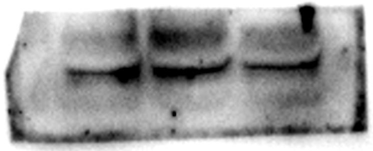

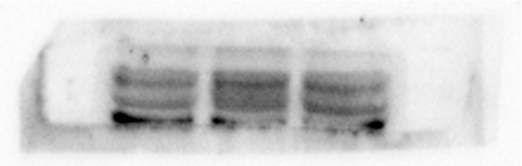

βactin

Control T1DM AD-1 Control T1DM AD-1 Control T1DM AD-1

45KD

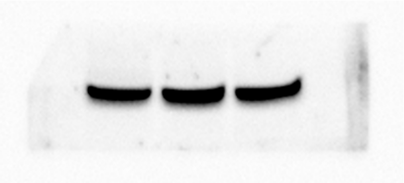

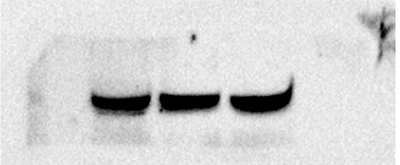

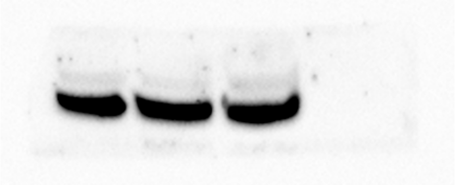

Supplement: Supplementary file 1 [file ijms-26-05300-s001.zip › ijms-3644474-supplementary.pdf]
